# Differential properties of human ACL and MCL stem cells may be responsible for their differential healing capacity

**DOI:** 10.1186/1741-7015-9-68

**Published:** 2011-06-02

**Authors:** Jianying Zhang, Tiffany Pan, Hee-Jeong Im, Freddie H Fu, James HC Wang

**Affiliations:** 1 MechanoBiology Laboratory, Departments of Orthopedic Surgery and Bioengineering, University of Pittsburgh, PA 15213, USA; 2 Departments of Biochemistry and Internal Medicine, Rush University Medical Center, Chicago, IL 60612, USA

## Abstract

**Background:**

The human anterior cruciate ligament (hACL) and medial collateral ligament (hMCL) of the knee joint are frequently injured, especially in athletic settings. It has been known that, while injuries to the MCL typically heal with conservative treatment, ACL injuries usually do not heal. As adult stem cells repair injured tissues through proliferation and differentiation, we hypothesized that the hACL and hMCL contain stem cells exhibiting unique properties that could be responsible for the differential healing capacity of the two ligaments.

**Methods:**

To test the above hypothesis, we derived ligament stem cells from normal hACL and hMCL samples from the same adult donors using tissue culture techniques and characterized their properties using immunocytochemistry, RT-PCR, and flow cytometry.

**Results:**

We found that both hACL stem cells (hACL-SCs) and hMCL stem cells (hMCL-SCs) formed colonies in culture and expressed stem cell markers nucleostemin and stage-specific embryonic antigen-4 (SSEA-4). Moreover, both hACL-SCs and hMCL-SCs expressed CD surface markers for mesenchymal stem cells, including CD44 and CD90, but not those markers for vascular cells, CD31, CD34, CD45, and CD146. However, hACL-SCs differed from hMCL-SCs in that the size and number of hACL-SC colonies in culture were much smaller and grew more slowly than hMCL-SC colonies. Moreover, fewer hACL-SCs in cell colonies expressed stem cell markers STRO-1 and octamer-binding transcription factor-4 (Oct-4) than hMCL-SCs. Finally, hACL-SCs had less multi-differentiation potential than hMCL-SCs, evidenced by differing extents of adipogenesis, chondrogenesis, and osteogenesis in the respective induction media.

**Conclusions:**

This study shows for the first time that hACL-SCs are intrinsically different from hMCL-SCs. We suggest that the differences in their properties contribute to the known disparity in healing capabilities between the two ligaments.

## Background

The human anterior cruciate ligament (hACL) and medial collateral ligament (hMCL) are two major ligaments that function to stabilize the knee joint. Because knee joints are subjected to large mechanical loads, particularly in athletic settings, both ligaments are frequently injured. It has been established that the injured hACL rarely heals, often requiring surgical reconstruction. As a result, patients with injured ACLs typically experience recurrent instability of the knee joint [1], which could lead to development of osteoarthritis [2]. On the other hand, the injured hMCL typically heals with conservative, non-operative treatment [3,4].

Several theories have been proposed as to why this difference in healing capability exists between the ACL and MCL. These include intra-articular versus extra-articular environments, different mechanical environments [5,6], and differences in nitric oxide synthesis [7], vascular supply [8], and proliferative potential of fibroblasts [9,10]. In recent years, however, the importance of adult stem cells (ASCs) in tissue healing has been noted [11-13]. ASCs are characterized *in vitro *by their remarkable abilities to proliferate extensively in an uncommitted state (self-renewal) and differentiate into cell types of various tissue lineages (multi-potential), including adipocytes, chondrocytes, and osteocytes. ASCs are responsible for repair and regeneration of injured tissues by proliferation and differentiation. Multipotent ASCs have been found in various types of tissues including bone marrow [14], adipose tissue [15,16], umbilical cord [17], synovium [18], spinal cord [19], dental pulp [20], and periodontal ligaments [21]. Recently, human, mouse, and rabbit tendons were found to contain stem cells, and these tendon stem cells (TSCs) exhibit the three universal characteristics of ASCs: clonogenicity, self-renewal, and multi-differentiation potential [22,23]. Therefore, we inferred that hACL and hMCL also contain ASCs. Indeed, a previous study showed that cells derived from young rabbit ACLs and MCLs exhibit stem cell properties [24].

Because ASCs are responsible for repair and regeneration of injured tissues, and because injured ACLs and MCLs have differential healing capacities as noted above, we hypothesized in this study that both human ACLs and MCLs contain ASCs, but that they exhibit distinctive, ligament-specific properties. To test this hypothesis, we derived stem cells from normal human ACL and MCL samples from the same donors. We then characterized and compared the properties of the two types of ligament stem cells, denoted hACL-SCs and hMCL-SCs, respectively. Herein we report the findings of this study.

## Methods

### hACL and hMCL stem cell cultures

Human ACL and MCL tissue samples free of pathology were obtained from six adult donors ranging in age from 20 to 36 years old (Table 1). The protocol for obtaining the ligament tissue samples was approved by the University of Pittsburgh Institutional Review Board. To prepare the tissue cultures, the ligament sheath was removed to obtain the core portion of the ligament, which was then minced into small pieces, and each 100 mg of wet tissue samples were digested in 1 ml of PBS containing 3 mg of collagenase type I and 4 mg of dispase as described previously [23]. Single cell suspensions were cultured in either a 96 well plate (1 cell/well) or T25 flasks (4 × 10^5^/flask). After eight to ten days in culture, hACL-SCs and hMCL-SCs formed distinct colonies on the plastic surfaces of the plates or flasks. The colonies were visualized with methyl violet and then counted with a hemocytometer.

**Table 1:** Human ACL and MCL samples

Donor	Age	Race	Gender
1	26	Caucasian	M
2	27	Black	M
3	26	Black	F
4	36	Caucasian	M
5	20	Hispanic	M
6	29	Black	F

Trypsin was locally applied to each colony under microscopic visualization in order to detach stem cell colonies, and detached cells were collected and transferred to T25 flasks for further culture. The growth medium consists of Dulbecco's modified Eagle's medium (DMEM; Lonza, Walkersville, MD) supplemented with 20% fetal bovine serum (FBS; Atlanta Biologicals, Lawrenceville, GA), 100 μM 2-mercaptoethanol (Sigma-Aldrich, St Louis), 100 U/ml penicillin, and 100 μg/ml streptomycin (Atlanta Biologicals, Lawrenceville, GA). To measure the proliferative capacities of hACL-SCs and hMCL-SCs, we used population doubling time (PDT) as an index. Briefly, hACL-SCs or hMCL-SCs were seeded in 6-well plates at a density of 6 × 10^4^/well and cultured with growth medium until confluence. The PDT is calculated by dividing the total culture time by the number of generations [23].

### Expression of stem cell markers by hACL-SCs and hMCL-SCs

Immunocytochemistry was used to assay for expression of the following stem cell markers: nucleostemin, Oct-4, STRO-1, and SSEA-4. To perform immunostaining, hACL-SCs or hMCL-SCs were seeded in 12-well plates at a density of 3.5 × 10^4^/well and cultured with growth medium for 3 days. The medium was then removed, and the cells were washed with PBS once. The stem cells were first fixed in 4% paraformaldehyde in phosphate-buffered saline (PBS) for 20 minutes. For nucleostemin and Oct-4 staining, this step was followed by washing with 0.1% Triton-X100 for 15 minutes. All cells were then blocked with 3% mouse serum for 1 hour. In the first antibody reaction, the stem cells were incubated with either mouse anti-human STRO-1 (1:400, Cat. #39-8401, Invitrogen, Carlsbas, CA), mouse anti-human SSEA-4 (1:400, Cat. #414000, Invitrogen, Carlsbas, CA), goat anti-human nucleostemin (1:350, Cat. # GT15050, Neuromics, Edina, MN), or rabbit anti-human Oct-3/4 (1:350, Cat. # sc-9081, Santa Cruz Biotechnology, Santa Cruz, CA) at room temperature for 2 hours. After washing the cells with PBS, cyanine 3 (Cy3)-conjugated goat anti-mouse immunoglobulin G (IgG) secondary antibody (1:500, Cat.# A10521, Invitrogen, Catlsbas, CA) was applied at room temperature for 1 hour to STRO-1 and SSEA-4 samples while Cy3-conjugated donkey anti-goat IgG secondary antibody (1:500, Cat.# AP180C, Millipore, Temecula, CA) was used for nucleostemin and Cy-3 conjugated goat anti-rabbit IgG antibody (1:400, Cat. # AP132C, Millipore, Temecula, CA) was used for Oct-3/4 samples at room temperature for 2 hours. The cells were also counterstained with Hoechst 33342 (Cat. # 33270; Sigma, St Louis).

In addition, stem cell surface markers CD31, CD44, CD90, CD34, CD45, and CD146 were stained in parallel by immunocytochemistry. Fixed cells were incubated with fluorescein isothiocyanate (FITC)- or Cy3- or phycoerythrin (PE)-conjugated mouse anti-human antibodies (1:400) for 1 hour. All steps were performed at room temperature. Unless otherwise noted, all antibodies were purchased from Chemicon International (Temecula, CA), BD Pharmingen (BD Biosciences; http://bdbiosciences.com), or Santa Cruz Biotechnology (Santa Cruz, CA). Fluorescent images of the stained cells were taken by a CCD camera on an inverted fluorescent microscope (Nikon eclipse, TE2000-U) using SPOT™ imaging software (Diagnostic Instruments, Inc., Sterling Heights, MI).

### Multi-differentiation potentials of hACL-SCs and hMCL-SCs

The multi-differentiation potentials of hACL-SCs and hMCL-SCs were examined *in vitro *to determine whether they could undergo adipogenesis, chondrogenesis, and osteogenesis. Cells at passage 1 were seeded in a 6-well plate at a density of 2.4 × 10^5 ^cells/well in basic growth medium consisting of low glucose DMEM, 10% heat inactivated FBS, 100 U/ml penicillin, and 100 μg/ml streptomycin. After reaching confluence, cells for adipogenesis were cultured in adipogenic induction medium (Millipore, Cat. # SCR020) consisting of basic growth medium supplemented with 1 μM dexamethasone, 10 μg/ml insulin, 100 μM indomethacin, and 0.5 mM isobutylmethylxanthine (IBMX) for 21 days. Oil Red O assay was used to detect lipid droplets contained in the differentiated adipocytes.

For chondrogenesis, confluent stem cells were cultured in chondrogenic induction medium consisting of basic growth medium along with 40 μg/ml proline, 39 ng/ml dexamethasone, 10 ng/ml transforming growth factor beta 3 (TGF-β3), 50 μg/ml ascorbate 2-phosphate, 100 μg/ml sodium pyruvate, and 50 mg/ml ITS premix (BD, Cat. # 354350). After 21 days in culture, the glycosaminoglycans (GAG)-rich matrix produced by differentiated chondrocytes was stained using the Safranin O assay.

Finally, for osteogenic differentiation, stem cells were cultured in osteogenic induction medium consisting of basic growth medium with 0.1 μM dexamethasone, 0.2 mM ascorbic 2-phosphate, and 10 mM glycerol 2-phosphate for 21 days. The differentiated cells released calcium-rich deposits, which were stained by the Alizarin Red S assay. Cells cultured in basic growth medium for the same durations were used as a control.

### Oil Red O assay

After discarding the medium, the cells were washed 3 times for 5 minutes each with PBS. The cells were then fixed in 4% paraformaldehyde for 40 minutes at room temperature. Subsequently, the cells were washed 3 times with PBS at 5 minute intervals and then with water twice for 5 minutes each. Finally, the cells were incubated with 0.36% Oil Red O solution (Millipore, Cat. # 90358) for 50 minutes, followed by washing 3 times with water.

### Safranin O assay

The cells were fixed in ice-cold ethanol for 1 hour, rinsed with distilled water twice for 5 minutes each, and stained with Safranin O solution (Sigma, Cat. # HT904) for 30 minutes. The cells were then rinsed five times with distilled water.

### Alizarin Red S assay

The cells cultured in osteogenic medium were fixed in chilled 70% ethanol for 1 hour, rinsed with distilled water for 5 minutes twice, and stained with Alizarin Red S solution (Millipore, Cat. # 2003999) for 30 minutes at room temperature. The cells then underwent five rinses with distilled water. Cells stained with the three assays were examined, and images were taken and analyzed on an inverted fluorescent microscope as noted earlier.

### Semi-quantification of the extent of cell differentiation

Briefly, 12 views from each well were randomly chosen on a microscope with a magnification of 20×. Then, the areas of positive staining were identified manually and computed by SPOT IMAGING Software. Next, the proportion of positive staining was calculated by dividing the stained area by the view area. Twelve ratio values for each of three wells were averaged to obtain the percentage of positive staining, which represents the extent of cell differentiation in the respective induction medium.

### Flow cytometry (FACS) analysis

The cell suspension (2.5 × 10^6 ^in 50 μl PBS) was incubated with 20 μl of the appropriate serum in a round-bottomed tube at 4°C for 30 minutes. Subsequently, 2 μl of primary antibody (0.2 mg/ml stock solution) was added and incubated at 4°C for 1 hour. The cells were then washed three times with 2% FBS-PBS and reacted with 1 μl of secondary antibody (1 mg/ml stock solution) at 4°C for 1 hour. Afterwards the cells were washed twice with PBS and then fixed with 0.5 ml of 1% paraformaldehyde. FACS analysis was performed with BD LSR II Flow Cytometer (BD Biosciences, http://www.bdbiosciences.com).

### Gene expression analysis by RT-PCR

RNA was extracted from hACL-SCs and hMCL-SCs using the RNeasy Mini Kit with an on-column DNase I digest (Qiagen). First-strand cDNA was synthesized from 1 μg total RNA, which was synthesized in a 20 μl reaction by reverse transcription using SuperScript II (Invitrogen). The following conditions for cDNA synthesis were applied: 65°C for 5 minutes and cooling for 1 minute at 4°C, then 42°C for 50 minute and 72°C for 15 minute. Next, qRT-PCR was performed using QIAGEN QuantiTect SYBR Green PCR Kit (QIAGEN). In a 50 μl PCR reaction mixture, 2 μl cDNA (total 100 ng RNA) were amplified in a Chromo 4 Detector (MJ Research, Maltham, MA) with incubation at 94°C for 5 minutes, followed by 30 to 40 cycles of a three temperature program of 1 minute at 94°C, 30 seconds at 57°C, and 30 seconds at 72°C. The PCR reaction was terminated after a 10 minute extension at 70°C and stored at 4°C until analysis. The following human-specific primers based on previous publications were used: Oct-4, STRO-1, peroxisome proliferator-activated receptor gamma (PPARγ, lipoprotein lipase (LPL), Sox-9, collagen type II (Coll. II), Runx2, and alkaline phosphatase (ALP). Glyceraldehyde 3-phosphate dehydrogenase (GAPDH) served as an internal control (Table 2). All primers were synthesized by Invitrogen. The products (each 5 μl) from qRT-PCR were run on a 2% agarose gel in 0.5×TBE buffer at 100 V. The separated DNA fragments were analyzed by a gel documentation system (Bio-Rad).

**Table 2:** Primers used for RT-PCR analysis

*Gene*	*Primer sequence*	*Reference*
PPAR_Y_	5'-GCT GTT ATG GGT GAA ACT CTG-3' 5'-CTC GGA CGT AGA GGT GGA ATA-3'	Risbud MV, et al. 2007
LPL	5'-GAG ATT TCT CTG TAT GGC ACC-3' 5'-CTG CAA ATG AGA CAC TTT CTC-3'	Risbud MV, et al. 2007
SOX-9	5'-ATC TGA AGA AGG AGA GCG AG-3' 5'-TCA GAA GTC TCC AGA GCT TG-3'	Risbud MV, et al. 2007
Collagen II	5'-TTT CCC AGG TCA AGA TGG TC-3' 5'-TCA CCT GGT TTT CCA CCT TC-3'	Risbud MV, et al. 2007
Runx2	5'-ACG ACA ACC GCA CCA TGG T-3' 5'-CTG TAA TCT GAC TCT GTC CT-3'	Risbud MV, et al. 2007
ALP	5'-TGG AGC TTC AGA AGC TCA ACA CCA-3' 5'-ATC TCG TTG TCT GAG TAC CAG TCC-3'	Risbud MV, et al. 2007
Oct-4	5'-GTG GTG GTA CGG GAA ATC AC-3' 5'-TAG CCA GGT TCG AGA ATC CA-3'	Huangfu D, et al. 2008
STRO-1	5'-GAA GCT AAA GTG GAT TCA GGA GTA-3' 5'-TAA GCA GGG GAC CAT TAC A-3'	Rada T, et al. 2010
GAPDH	5'-GGG CTG CTT TTA ACT CTG GT-3' 5'-TGG CAG GTT TTT CTA GAC GG-3'	Risbud MV, et al. 2007

### Data Analysis

For each experimental condition, at least three replicates were performed. The results presented in the figures are representative of these (mean ± SD, n = 3 to 6). Two-tailed student *t*-test was used for statistical analysis. A *P*-value less than 0.05 was considered to be significantly different.

## Results

### Clonogenicity and growth capacity of hACL-SCs and hMCL-SCs

**Figure 1:**
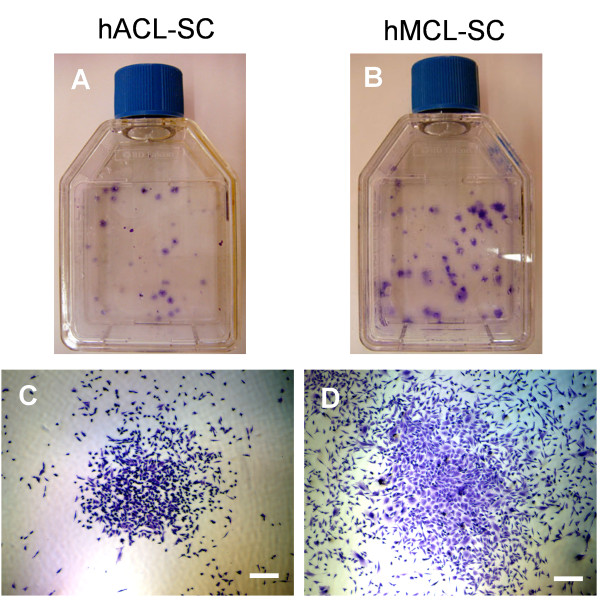
**Colony formation by hACL-SCs and hMCL-SCs**. **A**. hACL-SC colonies. **B**. hMCL-SC colonies. It is evident that hACL-SCs formed fewer colonies than hMCL-SCs. **C**. A sample colony of hACL-SCs. **D**. A sample colony of hMCL-SCs. Notably, the hACL-SC colony is much smaller than the hMCL-SC colony. Note that the results shown here were obtained from a 27-year-old male donor. (Bar: 100 μm).

**Figure 2:**
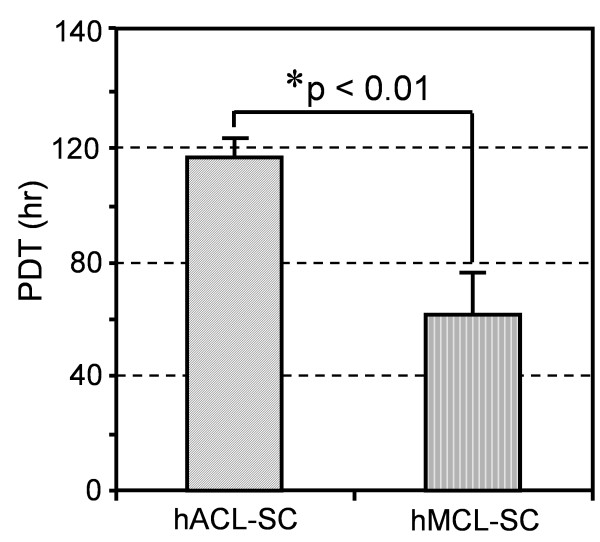
**The population doubling times (PDTs) of hACL-SCs and hMCL-SCs**. The PDT of hACL-SCs was markedly greater than that of hMCL-SCs, indicating that hACL-SCs proliferated more slowly than hMCL-SCs. The PDT results were obtained from passage 1 to passage 2 for both ACL-SCs and MCL-SCs from six donors (see Table 1).

After three days in culture, cells from single cell suspensions of both hACL and hMCL tissue samples attached to plate surfaces and formed colonies. However, the number and size of cell colonies from hACL-SCs and hMCL-SCs were markedly different: colonies formed by hACL-SCs were fewer in number (Figure 1A, B) and smaller in size than those of hMCL-SCs (Figure 1C, D). Moreover, hACL-SCs grew much more slowly than hMCL-SCs, as the PDT for hACL-SCs was nearly double that of hMCL-SCs (Figure 2).

### Stem cell marker expression of hACL-SCs and hMCL-SCs

**Figure 3:**
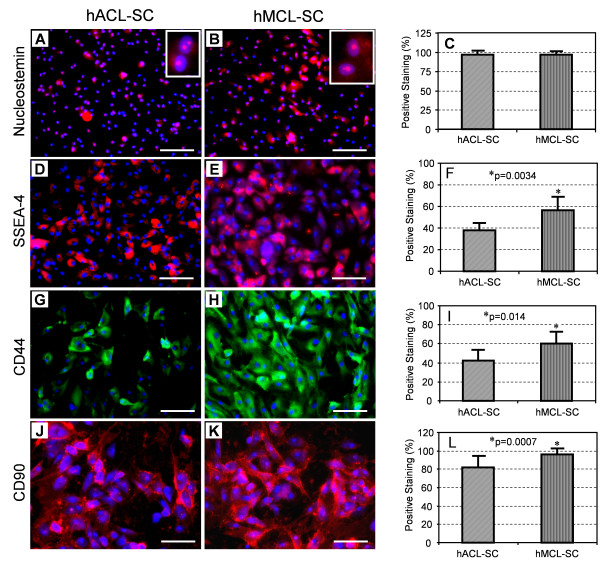
**The expression of stem cell markers in hACL-SCs and hMCL-SCs**. Both types of ligament stem cells expressed nucleostemin (**A, B, C**), SSEA-4 (**D, E, F**), CD44 (**G, H, I**), and CD90 (**J, K, L**), but not CD31, CD34, CD45, and CD146 (not shown). Note that negative controls (omission of primary antibodies) were also used in the immunostaining, and no staining signals were seen (data not shown). Also, the results shown here were obtained from a of 26-year-old male donor (see Table 1). The passage 1 cells were used in immunostaining. (Bar: 100 μm).

Using immunocytochemistry, both hACL-SCs and hMCL-SCs were found to express nucleostemin (Figure 3A, B), SSEA-4 (Figure 3D, E), CD44 (Figure 3G, H), and CD90 (Figure 3J, K). There was no significant difference in nucleostemin expression between hACL-SCs and hMCL-SCs, and more than 95% of both stem cells stained positively for nucleostemin (Figure 3C). However, only 40% of hACL-SCs were positively stained by SSEA-4, whereas more than 56% of hMCL-SCs were positive stained (Figure 3F). Similarly, about 42% of hACL-SCs expressed CD44 compared to about 60% for hMCL-SCs (Figure 3I). Furthermore, both hACL-SCs and hMCL-SCs expressed high levels of CD 90 (Figure 3L). Immunostaining for CD31, CD34, CD45, and CD146 was negative (data not shown).

**Figure 4:**
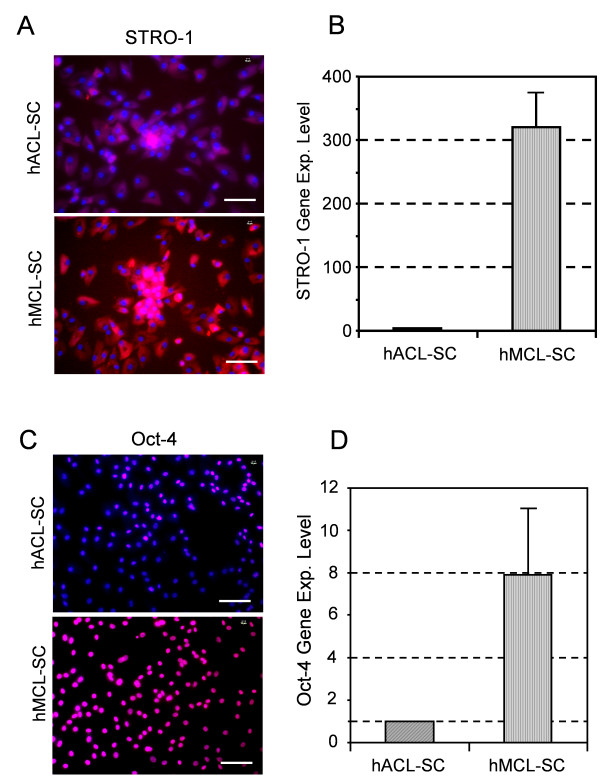
**The expression of two stem cell markers STRO-1 and Oct-4**. As seen, hACL-SCs stained more weakly for STRO-1 than hMCL-SCs (**A**). In addition, qRT-PCR showed that hACL-SCs expressed more than 300-fold lower levels of the STRO-1 gene than hMCL-SCs (**B**). Similarly, fewer hACL-SCs expressed Oct-4 than hMCL-SCs (**C**), and Oct-4 gene expression by hACL-SCs was more than 7 times lower than that of hMCL-SCs (**D**). Note that the results shown here were obtained from a 20-year-old male donor (see Table 1). The passage 1 cells were used in immunostaining. (Bar: 100 μm).

Moreover, hACL-SCs stained weakly for STRO-1, whereas more than 95% of hMCL-SCs stained positive for STRO-1 (Figure 4A). The gene expression level of STRO-1 in hACL-SCs was much lower than in hMCL-SCs (Figure 4B). Similarly, fewer than 40% of hACL-SCs expressed Oct-4, but more than 90% of hMCL-SCs stained positive for Oct-4 (Figure 4C). Finally, hACL-SCs expressed much lower levels of the Oct-4 gene than hMCL-SCs (Figure 4D).

**Figure 5:**
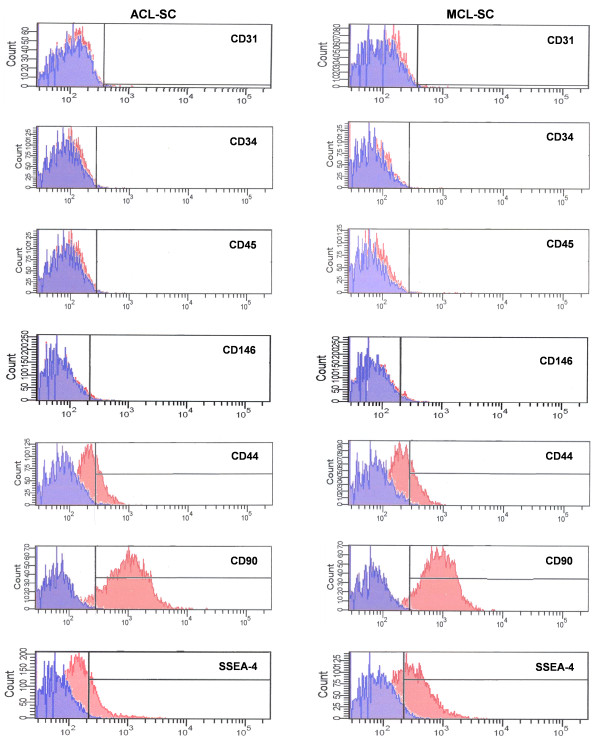
**FACS analysis of hACL-SCs and hMCL-SCs**. Both ligament stem cells were labeled with antibodies against human surface antigens as listed. The maximum positive count for CD31, CD34, CD45, and CD146 was less than 2%, and there was no significant difference between hACL-SCs and hMCL-SCs. However, large percentages (> 20%) of both ligament stem cells expressed CD44, CD90, and SSEA-4. The extent of expression of these stem cell markers by hACL-SCs and hMCL-SCs was significantly different (see Table 3). Note that hACL-SCs and hMCL-SCs (passages 1-2) used in the FACS analysis were obtained from four to six donors.

In addition, FACS analysis results showed that the percentages of CD31, CD34, CD45, and CD146 positive cells were less than 2%. Moreover, while CD44, CD90, and SSEA-4 were expressed to a greater extent by both hACL-SCs and hMCL-SCs (Figure 5), there was a significant difference in the level of expression between the two types of stem cells (Table 3).

**Table 3:** FACS results of stem cell marker expression (%)

Marker	SSEA-4	CD44	CD90
hACL-SC	20.1 ± 1.2	23.8 ± 5.8	89.1 ± 0.9

hMCL-SC	62.4 ± 6.0	48.7 ± 5.7	94.6 ± 1.3

*P*-value	0.002	0.006	0.002

### Self-renewal of hACL-SCs and hMCL-SCs

Both hACL-SCs and hMCL-SCs were able to undergo self-renewal, indicated by the maintenance of a cobblestone shape after repetitive passage and expression of stem cell markers nucleostemin and SSEA-4.

**Figure 6:**
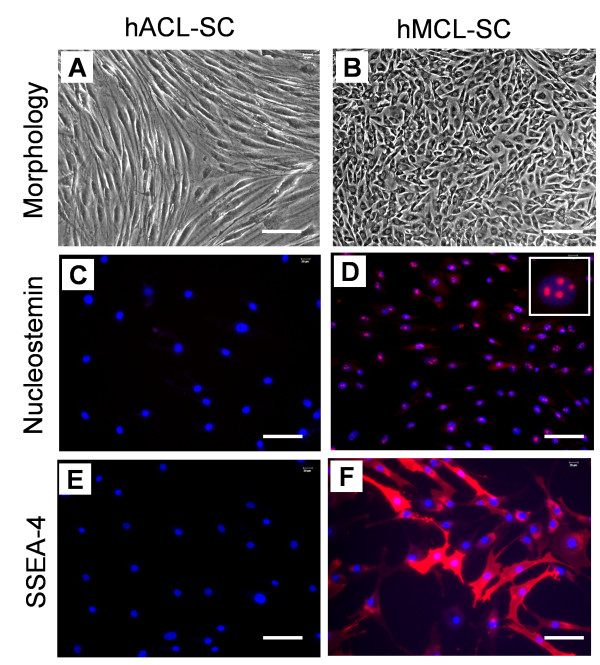
**Self-renewal of hACL-SCs and hMCL-SCs**. At passage 5, hACL-SCs had already become highly elongated in confluent culture, a typical fibroblast phenotype (**A**). In contrast, even at passage 13, confluent hMCL-SCs remained cobblestone-like (**B**). Moreover, hACL-SCs no longer expressed nucleostemin (**C**) or SSEA-4 (**E**) at passages > 5, whereas hMCL-SCs expressed both stem cell markers at passage 13 (**D, F**). Note, however, that hMCL-SCs at this high passage exhibited a lesser degree of nucleostemin expression compared to the cells at passage 1 (see Figure 3). The results shown here were obtained from a male donor of 27 years old (see Table 1). (Bar: 100 μm).

However, after five passages and two months in culture, hACL-SCs became elongated (Figure 6A), a typical fibroblast phenotype, and lost expression of nucleostemin and SSEA-4 (Figure 6C, E), indicating that they had undergone differentiation. In contrast, hMCL-SCs, even after 13 passages and two months of culture time, maintained a cobblestone shape (Figure 6B) and expressed a high level of nucleostemin and SSEA-4 (Figure 6D, F). The extent of nucleostemin expression at this passage, however, was lower than that at passage 1 (Figure 3).

### Multidifferentiation potential of hACL-SCs and hMCL-SCs

**Figure 7:**
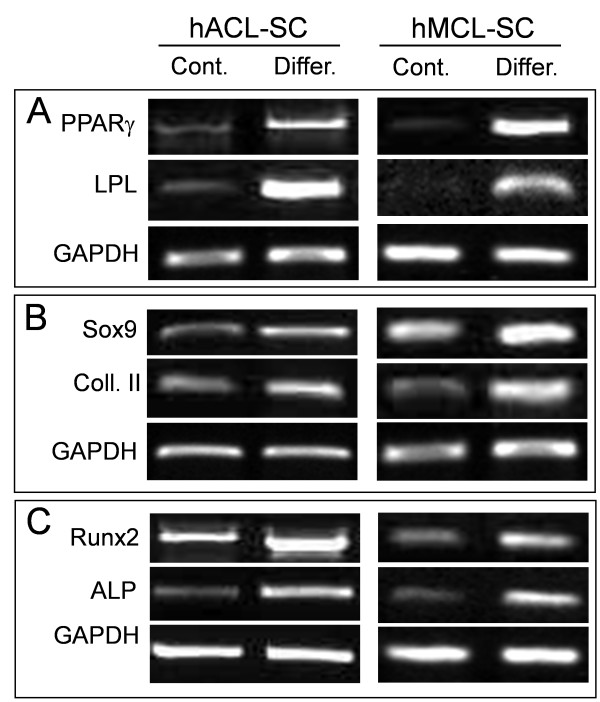
**The expression of marker genes for adipogenesis (A), chondrogenesis (B), and osteogenesis (C)**. Compared to control cells, these marker genes in both hACL-SCs and hMCL-SCs were highly upregulated when the ligament stem cells were grown in the respective induction media. The RT-PCR results were obtained from a 29-year-old female donor (see Table 1). The results from another two donors were similar (not shown). ALP, alkaline phosphatase; Coll. II, collagen type II; Cont., control; Differ., differentiated; LPL, lipoprotein lipase;.

After 21 days in adipogenic media, both hACL-SCs and hMCL-SCs expressed high levels of PPARγ and LPL (lipoprotein lipase), two adipogenesis markers, indicating that the cells had differentiated into adipocytes (Figure 7A). When grown in chondrogenic media, these ligament stem cells differentiated into chondrocytes, evidenced by upregulation of Sox-9 and Collagen type II expression (Figure 7B), which are two markers for chondrogenesis. Finally, both hACL-SCs and hMCL-SCs in osteogenic media differentiated into osteocytes, as two osteogenesis markers Runx2 and ALP were significantly upregulated (Figure 7C).

**Figure 8:**
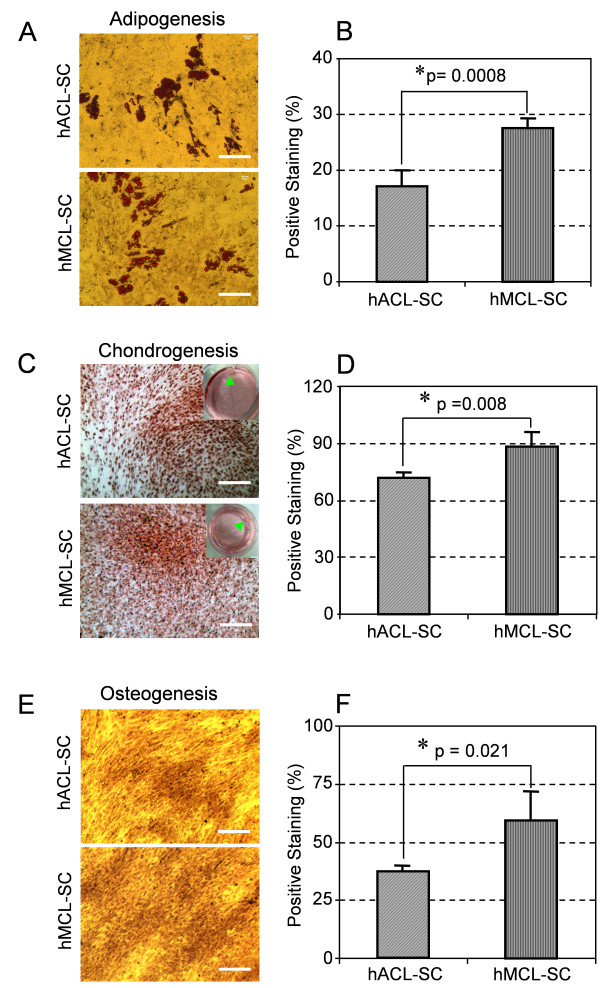
**Histochemical staining of differentiated cells and semi-quantification of the extent of cell differentiation**. Both hACL-SCs and hMCL-SCs were able to differentiate into adipocytes (**A**), chondrocytes (**C**), and osteocytes (**E**), as shown by the accumulation of lipid droplets, proteoglycans, and calcium deposits on cell surfaces. However, the extent of hACL-SC differentiation was less than that of hMCL-SC differentiation, evidenced by smaller positive staining areas for adipogenesis (**B**), chondrogenesis (**D**), and osteogenesis (**F**) in hACL-SCs than in hMCL-SCs. Note that each experiment was repeated three times using three different donors. (Bar: 100 μm).

Using respective histochemical staining, we further demonstrated that both hACL-SCs and hMCL-SCs differentiated into adipocytes, chondrocytes, and osteocytes in the respective induction medium, evidenced by formation of lipid droplets (Figure 8A), glycosaminoglycans (GAG)-rich matrix (Figure 8C), and calcium-rich deposits (Figure 8E). Of note is that these stem cells could form cartilage-like pellets in chondrogenic induction medium (insets, Figure 8C). Semi-quantification of stained areas showed that there were significant differences in the extent of adipogenesis (Figure 8B), chondrogenesis (Figure 8D), and osteogenesis (Figure 8F) between hACL-SCs and hMCL-SCs.

## Discussion

Adult stem cells were isolated and identified from hACLs and hMCLs. We showed that these hACL-SCs and hMCL-SCs exhibit characteristic stem cell properties, including clonogenicity, self-renewal, and multi-potency. Furthermore, both populations expressed stem cell markers nucleostemin, SSEA-4, STRO-1, and Oct-4 as well as several CD markers (CD44 and CD90) for mesenchymal stem cells (MSCs), but not those for endothelial cells, hematopoietic stem cells, leukocytes, or pericytes (CD31, CD34, CD45, and CD146). However, it was found that a smaller proportion of hACL-SCs expressed STRO-1, Oct-4, and CD44 compared to hMCL-SCs. hACL-SCs also grew about 50% slower and formed smaller and fewer colonies than hMCL-SCs. Moreover, there was a marked difference in long-term self-renewal capability between the two types of stem cells: hACL-SCs became differentiated after only five passages and two months in culture, whereas hMCL-SCs maintained a nearly undifferentiated state even after 13 passages and the same culture time. Taken together, these results show that hACL-SCs and hMCL-SCs are ligament specific stem cells that possess intrinsically different stem cell properties.

Nucleostemin, SSEA-4, STRO-1, and Oct-4 are four well established stem cell markers used to confirm the stem cell identity of hACL-SCs and hMCL-SCs in this study. Nucleostemin is a nucleolar protein believed to act via p53 [25,26] and to be expressed by stem cells and cancer cells but not terminally differentiated cells [26,27]. Thus, high levels of nucleostemin expression by hACL-SCs and hMCL-SCs in this study were indicative of proliferating, self-renewing populations of ASCs. Like nucleostemin, Oct-4 is also a nuclear protein expressed in embryonic stem cells and carcinoma cell lines but not in differentiated cells [28]. Oct-4 expression is lost during the process of differentiation, and downregulation of Oct-4 is thought to directly induce stem cell differentiation [29-31].

SSEA-4 is a member of the stage specific antigen family first identified as a marker that disappeared from human teratocarcinoma cells as they differentiated [32,33], which has since been recognized as a marker of human embryonic stem cells [34] and mesenchymal stem cells [35] as well. STRO-1 is a cell surface antigen found on bone marrow mononuclear cells [36] capable of differentiating down osteogenic [37,38], chondrogenic, and adipogenic lines [39]. In addition, STRO-1 is expressed in human periodontal ligament stem cells [40].

In addition to the stem cell markers above, we examined the expression of CD surface markers on hACL-SCs and hMCL-SCs. Both ACL-SCs and MCL-SCs expressed CD44 and CD90 (albeit the former exhibited a less extent than the latter). Neither of the two types of ligament stem cells expressed CD31, CD34, CD45, or CD146. CD44 is a common MSC antigen [41,42] and is used as a marker for bone marrow stem cells (BMSCs) [22]. CD90 is a fibroblast marker that has also been found on undifferentiated human embryonic stem cells [43], and human MSCs are consistently positive for both CD44 and CD90 [44-46]. Endothelial cell marker CD31 [47], hematopoietic stem cell marker CD34 [48], pericyte marker CD146 [49], and leukocyte marker CD45 [50] were expressed by neither hACL-SCs nor hMCL-SCs. These results provide additional evidence that hACL-SCs and hMCL-SCs are ASCs of mesenchymal origin.

A recent study by Cheng et al. looked into the possibility of populations of stem cells existing in human ACLs [51]. It was shown that cells isolated from the ACL are clonogenic with multidifferentiation potential and express surface markers similar to MSCs, including CD73, CD90, and CD105. The hACL-SCs isolated in our study displayed similar characteristics as the ligament stem cells from Cheng et al.'s study in terms of clonogenicity, multipotency, and expression of stem cell markers CD44 and CD90, but not CD34 or CD45. However, unlike this study, the ACL samples used by Cheng et al. may not be normal as they were collected from patients who had undergone total knee arthroplasty.

Another study, in which ligament cells were derived from young rabbits, found that the chondrogenic potential of 'ligament-derived cells' was greater for ACL cells than MCL cells [24]. However, their study used a mixed cell population rather than isolated stem cells as this study did. Finally, tissue specific stem cells, such as hACL-SCs and hMCL-SCs, have been found in various tissues, including bone marrow [14], the periodontal ligament [21], and human, mouse, and rabbit patellar tendons [22,23], which are similar to extra-articular ligaments. In addition, rat flexor tendons were found to contain stem cells [52]. Our group has also shown that stem cells from rabbit patellar and Achilles tendons express nucleostemin, Oct-4, and SSEA-4 [23]. The same stem cell markers were found to be expressed on both hACL-SCs and hMCL-SCs in this study.

While both hACL-SCs and hMCL-SCs were shown to be ASCs, differences were also observed with regard to their clonogenicity, self-renewal capacity, and differentiation potential. We previously also found that stem cells derived from rabbit patellar and Achilles tendons exhibit marked differences in colony formation and cell proliferation rate [23]. Differences in gene profiles have also been noted between MSCs derived from human intra-articular (synovium, meniscus, and ACL) and extra-articular sources (adipose, muscle, and bone) [42]. In addition, previous studies showed that ACL fibroblasts proliferate more slowly than their MCL counterparts [9,53]. These above studies, however, differ from this study in that they used mixed cultures; in other words, the cell population could include both stem cells and residential adult cells (for example, ACL fibroblasts). Nevertheless, the finding in this study that hACL-SCs proliferate much more slowly than hMCL-SCs (Figure 2) is largely consistent with conclusions of the above studies.

It is well recognized that injured ACLs have a low healing capacity, whereas injured MCLs display a high healing capacity [54-56]. Given that ASCs are the body's natural reservoir for replenishing pools of specialized cells that have been damaged in tissue injury, we suggest that the differential characteristics of hACL-SCs and hMCL-SCs found in this study may also contribute to their respective ligaments' differential healing capacities. Specifically, our data seem to indicate that hACL-SCs lose their 'stemness' earlier than hMCL-SCs. This may contribute to non-healing of injured ACLs as the hACL-SCs may have lost their ability to self-renew during the healing process; as a result, few cells will be available for repair of the injured ACLs. On the other hand, because of their superior capability of self-renewal, hMCL-SCs can continuously supply cells to effectively repair injured MCLs.

Besides the inherent lower stem cell capacity of hACL-SCs, blood flow, an 'external' factor, is known to be lower in the ACL than in the MCL in both intact and injured states. As a result, fewer nutrients will be available to hACL-SCs compared to hMCL-SCs. Therefore, again a smaller number of hACL-SCs and their progeny cells will be produced compared to hMCL-SCs.

The finding that the hACL contains ASCs may enable one to devise a new tissue engineering approach for repair of injured hACLs. This could be done by using small portions of ligaments to isolate and expand hACL-SCs *in vitro *and then implanting the cells into the injured ACL. On the other hand, while the injured MCL heals spontaneously, the quality of the healed tissue is still inferior with scar formation [57]. This is true even with implantation of natural scaffolding materials [58,59]. Therefore, hMCL-SCs may also be used as a source for cellular-based therapies to restore the structure and function of the injured MCL.

Several comments are now in place regarding the proper interpretation of the results of this study. First, we used local application of trypsin to isolate stem cell colonies in cultures. Such a technique may be subject to contamination of a small number of ligament fibroblasts; in other words, the stem cell populations used in this study may not be pure. Second, there is an apparent difference in the results of stem cell marker expression between immunocytochemistry (Figure 3) and FACS analysis (Table 3). The difference may be caused by the different passages of cells and the number of donors used in the two different methods. For immunostaining we used hACL-SCs and hMCL-SCs at passage 1 from a 26 year old donor, but for FACS analysis, passages 2 to 3 from six donors were used and the results were represented by mean ± SD. Third, hACL-SCs and hMCL-SCs were found to express low levels of non-tenocyte related genes, including PPARγ, LPL, Sox-9, collagen II, and Runx-2, even without differentiation induction media. There are two possible reasons for this. While the ligaments used in our experiments were limited at grade 0 (normal), the donors might have slight degenerative changes in their ACL and MCL ligaments. Additionally, there is a possibility that a small population of stem cells that somehow had differentiated towards non-fibroblasts was present in cultures.

## Conclusions

We show in this study that while both hACL-SCs and hMCL-SCs exhibited clonogenicity, self-renewal, and multi-differentiation potential, the three universal characteristics of ASCs, hACL-SCs differed from hMCL-SCs in that hACL-SCs expressed a much lower level of STRO-1 and Oct-4, two stem cell marker genes. Moreover, compared to hMCL-SCs, hACL-SCs exhibited a lower capacity for colony formation, slower proliferation, a shorter period of self-renewal capability, and a lower extent of multidifferentiation potential. As ASCs are responsible for repair and regeneration of injured tissues, we suggest that the differences in the properties of the two ligaments' stem cells may contribute to the differential healing capacities of injured ACLs and MCLs observed clinically.

## Abbreviations

ASCs: adult stem cells; BMSCs: bone marrow stem cells; Cy3: cyanine 3; FACS: fluorescence activated cell scanning; FITC: fluorescein isothiocyanate; GAPDH: glyceradehyde 3-phosphate dehydrogenase; GAG: glycosaminoglycans; hACL: human anterior cruciate ligament; hMCL: human medial collateral ligament; IgG: immunoglobulin G; MSCs: mesenchymal stem cells; Oct-4: octamer-binding transcription factor-4; PE: phycoerythrin; PPARγ: peroxisome proliferator-activated receptor gamma; SCs: stem cells; SSEA-4: stage-specific embryonic antigen-4; TGF-β3: transforming growth factor beta 3; TSCs: tendon stem cells.

## Competing interests

The authors declare that they have no competing interests.

## Authors' contributions

JZ performed experiments and assembled data. TP participated in experiments and assisted in drafting the manuscript. HJI helped to collect ligament samples. FF participated in initiation and discussion of the study. JHW designed the study, performed data analysis, and drafted the manuscript.

## Pre-publication history

The pre-publication history for this paper can be accessed here:

http://www.biomedcentral.com/1741-7015/9/68/prepub
